# Albumin–Hyaluronan Interactions: Influence of Ionic Composition Probed by Molecular Dynamics

**DOI:** 10.3390/ijms222212360

**Published:** 2021-11-16

**Authors:** Piotr Bełdowski, Maciej Przybyłek, Przemysław Raczyński, Andra Dedinaite, Krzysztof Górny, Florian Wieland, Zbigniew Dendzik, Alina Sionkowska, Per M. Claesson

**Affiliations:** 1 Faculty of Chemical Technology and Engineering, Institute of Mathematics & Physics, Bydgoszcz University of Science & Technology, 85-796 Bydgoszcz, Poland; 2 KTH Royal Institute of Technology, School of Engineering Sciences in Chemistry, Biotechnology and Health, Engineering Pedagogics, SE-100 44 Stockholm, Sweden; andra@kth.se; 3 Department of Physical Chemistry, Pharmacy Faculty, Collegium Medicum of Bydgoszcz, Nicolaus Copernicus University in Toruń, Kurpińskiego 5, 85-950 Bydgoszcz, Poland; m.przybylek@cm.umk.pl; 4 Faculty of Science and Technology, University of Silesia in Katowice, 75 Pułku Piechoty 1A, 41-500 Chorzow, Poland; przemyslaw.raczynski@us.edu.pl (P.R.); krzysztof.gorny@us.edu.pl (K.G.); zbigniew.dendzik@us.edu.pl (Z.D.); 5 Division of Bioscience and Materials, RISE Research Institutes of Sweden, SE-114 86 Stockholm, Sweden; 6 Helmholtz-Zentrum Hereon: Institute for metallic Biomaterials, Max-Planck-Straße 1, 21502 Geesthacht, Germany; florian.wieland@hereon.de; 7 Department of Biomaterials and Cosmetics Chemistry, Faculty of Chemistry, Nicolaus Copernicus University in Toruń, Gagarin 7, 87-100 Torun, Poland; alinas@umk.pl; 8 KTH Royal Institute of Technology, Department of Chemistry, Surface and Corrosion Science, School of Engineering Sciences in Chemistry, Biotechnology and Health, SE-100 44 Stockholm, Sweden; percl@kth.se

**Keywords:** hyaluronic acid, hyaluronan, human serum albumin, molecular dynamics simulations, hydrogen bonds, water mediated interactions, ionic interactions

## Abstract

The lubrication mechanism in synovial fluid and joints is not yet fully understood. Nevertheless, intermolecular interactions between various neutral and ionic species including large macromolecular systems and simple inorganic ions are the key to understanding the excellent lubrication performance. An important tool for characterizing the intermolecular forces and their structural consequences is molecular dynamics. Albumin is one of the major components in synovial fluid. Its electrostatic properties, including the ability to form molecular complexes, are closely related to pH, solvation, and the presence of ions. In the context of synovial fluid, it is relevant to describe the possible interactions between albumin and hyaluronate, taking into account solution composition effects. In this study, the influence of Na^+^, Mg^2+^, and Ca^2+^ ions on human serum albumin–hyaluronan interactions were examined using molecular dynamics tools. It was established that the presence of divalent cations, and especially Ca^2+^, contributes mostly to the increase of the affinity between hyaluronan and albumin, which is associated with charge compensation in negatively charged hyaluronan and albumin. Furthermore, the most probable binding sites were structurally and energetically characterized. The indicated moieties exhibit a locally positive charge which enables hyaluronate binding (direct and water mediated).

## 1. Introduction

Degenerative joint diseases including the most common osteoarthritis causing synovial inflammation, osteophyte, and other articular cartilage damage processes is a global health problem that affects millions of people around the world [1,2]. With the growing number of people suffering from obesity and aging populations, joint diseases are becoming increasingly common [1,3]. It has been estimated that osteoarthritis affects over 25% of the adult population [1]. From this point of view, a well working lubrication in an articular cartilage/synovial fluid system is important to maintain as this will ensure a high quality of life and low healthcare costs. However, the synovial fluid’s lubrication properties are strictly associated with the intermolecular interactions between the macromolecular and phospholipid components. The synovial fluid contains many diverse and important components, such as hyaluronan, phospholipids, and proteins such as γ-globulin, albumin, and lubricin that play major roles in the lubrication mechanism [4,5,6]. Albumin deserves special attention due to its binding and transporting properties of various compounds (fatty acids [7,8], bilirubin [9], steroids [10]) and ions, K^+^, Na^+^, and Mg^2+^ and Ca^2+^ [11,12,13,14,15,16,17,18,19].

The properties of albumin and γ-globulin are often compared in terms of their effect on lubrication. Murakami et al. [20] demonstrated that hyaluronic acid interacts with proteins found in the synovial fluid, like γ-globulin and albumin, which affects the tribological properties of cartilage. Interestingly, locally positively charged sites in albumin favoring interactions with the ionized carboxylic groups in hyaluronate should be expected to appear, even though both macromolecules have a global negative charge under the physiological conditions. 

In the work of Murakami et al. [20], the charge compensation with inorganic ionic species like Na^+^ (the lubricants were dissolved in 0.15 M NaCl) probably affected the complex stability. In the mentioned study, the authors analyzed the articular cartilage reciprocating tests using glass plates lubricated with hyaluronic acid, L-α-dipalmitoylphosphatidylcholine, γ-globulin, and albumin mimicking synovial fluid and porcine knee joint. It was established that the application of γ-globulin/hyaluronic acid resulted in lower restarting and final friction than pure hyaluronic acid. Interestingly, the albumin/hyaluronic acid system behaved somewhat differently since a higher final friction was observed. This was suggested to be due to the greater contribution of electrostatic repulsive forces between human serum albumin (HSA) and hyaluronic acid than between γ-globulin and the polysaccharide as judged from the isoelectric point of the proteins. HSA has an isoelectric point at pH ≈ 4.7, whereas it occurs at pH 7.2 for γ-globulin. Thus, both proteins are negatively charged in the synovial fluid with pH 7.6–8.2. Likewise, hyaluronic acid becomes deprotonated and is present in the form of negatively charged hyaluronate (HA). Thus, it was suggested that locally induced attractive forces could more easily stabilize γ-globulin/hyaluronan complexes [20,21,22,23] than HSA/hyaluronan complexes. Consistent with this, it has been shown that even when the pH is much higher than the albumin isoelectric point, attractive intermolecular interaction with hyaluronate can still be formed due to the presence of positively charged sites despite the overall negative charge of the whole protein [24,25,26,27]. Furthermore, the presence of albumin in combination with globulin and hyaluronic acid in joint cartilage models results in more effective lubrication compared to albumin-free systems [28,29]. These results are difficult to understand without further knowledge of the association and interaction between hyaluronan and these proteins. Moreover, the affinity of hyaluronate to proteins present in the synovial fluid cannot be considered without evaluating the effects of the inorganic components, such as water and especially dissolved ions which modify the electrostatic interactions in the system.

The effect of pH and sodium ions on the bovine serum albumin–hyaluronate system has been studied by Xu et al. [30] using dynamic and electrophoretic light scattering techniques and potentiometric measurements. It was demonstrated that there is a significant effect of the pH on the phase separation and the binding of de-protonated carboxyl groups of hyaluronan with albumin, and also resulted in the release of Na^+^ ions. This effect cannot be ignored when considering the interactions in the synovial fluid. The influence of an ion on the affinity of various ligands to albumin has been frequently studied using both experimental and theoretical methods. Some interesting examples are bovine serum albumin interactions in the presence of different cations with nutraceuticals such as tannic acid [31] and baicalein [32] and with drugs like zonisamide [33], efonidipine [34], and pentoxifylline [35].

**Figure 1:**
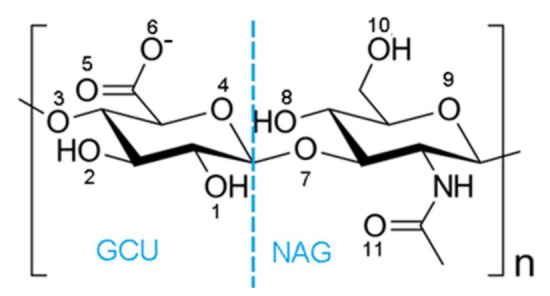
Structure of the repeating disaccharide unit of hyaluronate, the deprotonated form of hyaluronic acid. GCU stands for D-glucuronic acid with pKa of about 3, and NAG means N-acetyl-D-glucosamine. The different oxygen atoms are numbered, and this numbering will be utilized when discussing the interaction with human serum albumin.

**Figure 2:**
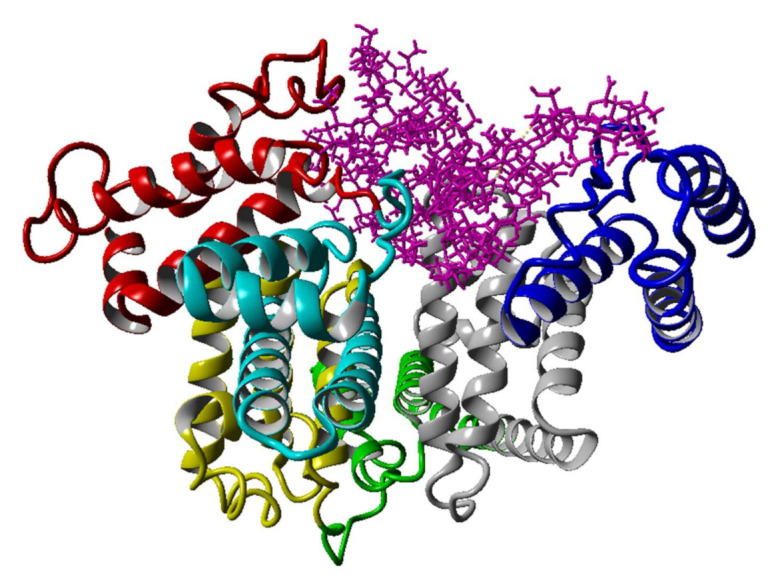
Structure of human serum albumin with different coloring for the different HSA subdomains: IA—red; IB—cyan; IIA—yellow; IIB—green; IIIA—grey; IIIB—blue. Hyaluronate is colored pink. The figure represents one of many structures of the HAS–hyaluronate complex. This particular complex is referred to as complex number 1 in Table 1.

The interaction between albumin and hyaluronate in the presence of various species such as water and ions is an interesting issue that is closely related to the unique properties of synovial systems. A second important reason for the interest in these systems is associated with the drug delivery enhancement abilities of various albumin–hyaluronan nanoparticles [26,27,36,37,38,39]. However, to the best of the authors knowledge there is very limited information about the structural features of albumin–hyaluronan molecular assemblies, including intermolecular interaction characteristics. The use of molecular modeling allows us to evaluate the influences of various factors, such as the presence of ions and solvation on the properties of proteins, including their ability to bind ligands. The aim of this study is to evaluate the effect of Na^+^, Mg^2+^, Ca^2+^ cations on the affinity of hyaluronate (Figure 1) to human serum albumin using molecular dynamics methods. Human serum albumin consists of a single chain of 585 amino acids, which incorporates three homologous domains (I, II, and III). Domain I consists of residues 5–197, domain II includes residues 198–382, and domain III is formed from residues 383–569. Each domain is composed of two sub-domains termed A and B (IA; residues 5–107, IB; residues 108–197, IIA; residues 198–296, IIB; residues 297–382, IIIA; residues 383–494, IIIB; residues 495–569), see Figure 2 for further details.

## 2. Results and Discussion

In order to generate the final structures enriched with appropriate cations and water molecules, the standard docking procedure was performed. Since docking gives only preliminary information on the stability of the structure, the obtained complexes were enriched with water molecules and subjected to molecular dynamics simulation. In this work, the affinity is expressed by the binding energy, which is the amount of energy that should be added to the system to remove the ligand from the receptor. The list of structures ranked according to increasing magnitude of binding energy calculated using molecular dynamics along with the docking ranks are summarized in Table 1. In Figure 2, the first structure listed in this ranking is presented (complex 1).

Based on the inspection of binding energy values calculated for different docking sites, it can be concluded that there are relatively small differences in the stability between the first two structures in the list characterized by the highest ligand–protein affinity. Complex 1 is characterized by about 6% higher binding energy value than the second structure on the list. Furthermore, they are somewhat structurally similar, since in case of both structures the hyaluronate interacts with the binding centers in a characteristic pocket formed by the IA, IB, IIIA, and IIIB subdomains. Notably, three of these albumin moieties (IB, IIIA, and IIIB) are regarded as key domains for the albumin transport function responsible for heme binding site (IB), Sudlow’s site II (IIIA), and thyroxine binding site (IIIB) [40]. Interestingly, the IB subdomain interacts with hyaluronate only in case of complexes 1 and 4. This is understandable, since IB is regarded to interact with highly non-polar hydrophobic compounds such as pyrene [41]. On the other hand, IIIA and IIIB are involved in all 12 assemblies determined through the docking procedure as best fitted complexes for the given docking algorithm. Interestingly, both fragments and in some cases IB are involved in the binding of non-steroidal molecules containing carboxylic groups, such as ibuprofen, ketoprofen, naproxen, diclofenac, and indomethacin [42,43,44]. Furthermore, it seems to be quite probable that the *S* configuration of hyaluronate carboxylic acid groups is beneficial for binding with the IIIA and IIIB subdomains, since (*S*)-enantiomers of 2-arylpropionic acids are capable of forming more stable interactions than (*R*)-enantiomers [42]. However, we note that the higher hydrophilicity of HA suggests a different nature of the binding of HA and acrylpropionic acid to albumin.

**Figure 3:**
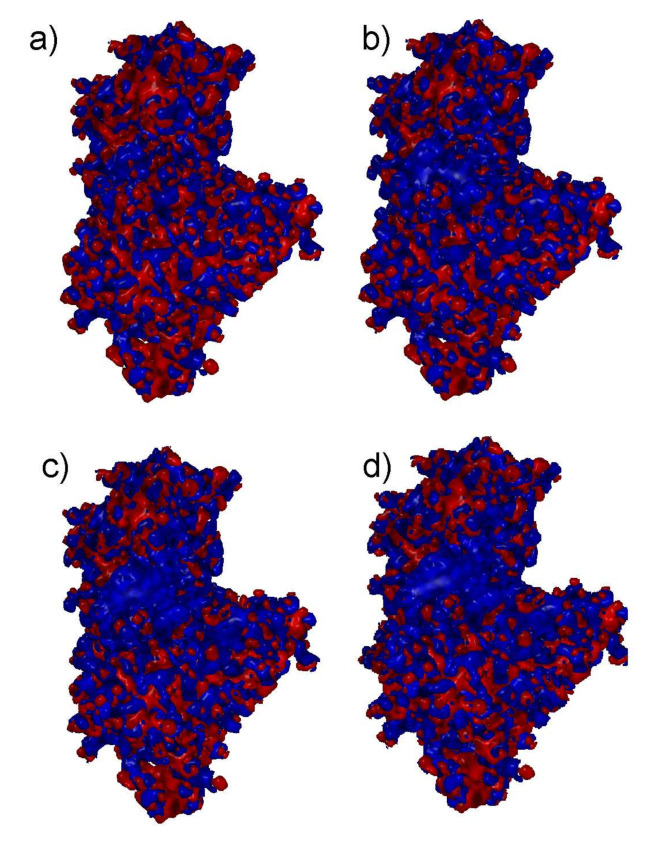
Electrostatic potential map of HSA where blue and red represent positively and negatively charged regions, respectively. Effects of different ions are presented: (**a**) no ions, (**b**) Na^+^, (**c**) Ca^2+^, (**d**) Mg^2+^.

Since the electrostatic interactions play a key role in the albumin–hyaluronate binding mechanism, an electrostatic potential map (Figure 3) was generated for the optimized albumin structure (with and without the addition of ions). When the presence of Na^+^, Mg^2+^, and Ca^2+^ cations is taken into account, a much higher positive charge density can be observed in the middle of the map (hyaluronate binding cavity). This observation is consistent with the binding mechanism of hyaluronic acid described in the literature [24,25,26,27], according to which, despite the globally negatively charged albumin molecule at physiological pH, there are positively charged parts that act as binding sites for the ligand.

**Figure 4:**
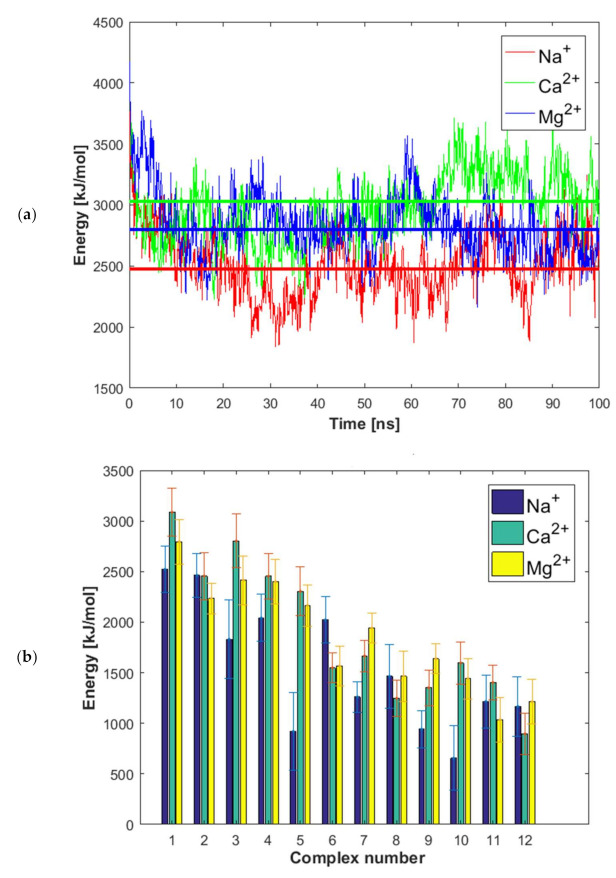
(**a**) HSA–HA binding energy vs. time average for complex 1 (constant line represents average over last 60 ns). (**b**) Binding energies for different complexes in presence of different cations for the simulation time of 40–100 ns. Complexes are sorted according to the average for all three ions.

In Figure 4a, the relationship between binding energy and simulation time is presented for complex number 1. As can be inferred from the natural fluctuations in binding energy, the complexes stabilization was reached within the applied simulation time. When analyzing Figure 4a, no increased stability of the albumin–hyaluronate complex in the presence of Mg^2+^ over that in the presence of Na^+^ can be observed after c.a. 70 ns. However, the presence of Ca^2+^ ions does increase the stability of the HSA–HA complex. The difference in the effect of Ca^2+^ and Mg^2+^ is suggested to be due to the lower hydration of Ca^2+^. Noteworthy, charge inversion and ion-bridge formation with divalent cations has been well described in the literature [45,46,47,48,49,50,51]. However, the obtained molecular dynamics simulations are not clear in the importance of these effects for the case of Mg^2+^.

**Figure 5:**
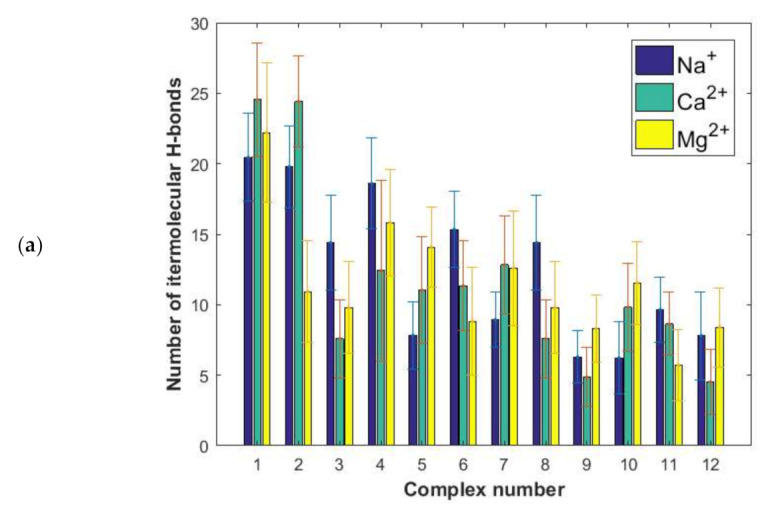

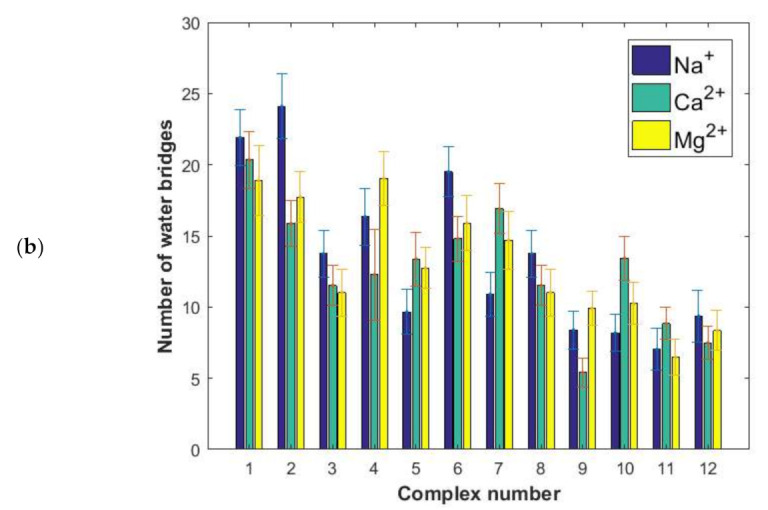
(**a**) Number of direct intermolecular H-bonds. (**b**) Water bridges between HSA and HA for different complexes evaluated by MD.

**Figure 6:**
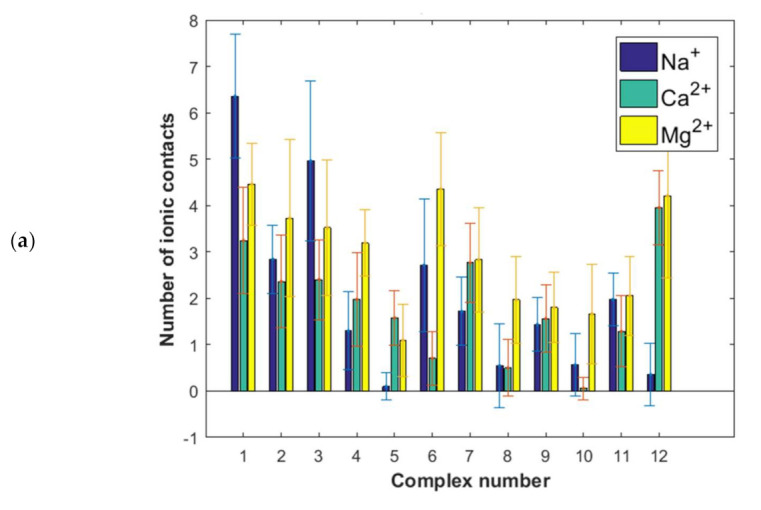

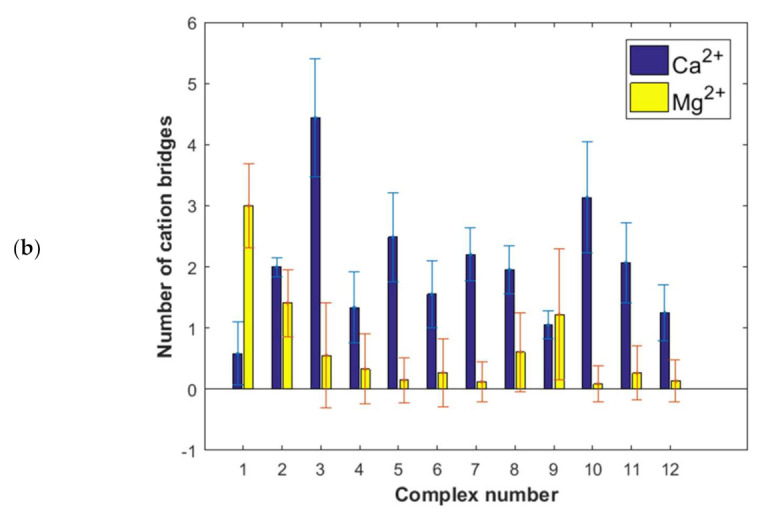
Number of ionic interactions between HSA and HA: (**a**) direct, (**b**) cation mediated (cation bridges).

We calculated the average binding energy values over the time domain 40–100 ns of the simulation, see Figure 4b, and the standard deviations reflect the range of the binding energy fluctuations. Complex 1 in the presence of Ca^2+^ was found to be characterized by the highest HAS-HA affinity. However, quite high affinity can also be observed for complex 3. By taking the binding energy fluctuations into account, complexes 1 and 3 are of similar energy. For 6 out of 12 complexes considered, the highest affinity of hyaluronan to albumin was observed in the presence of Ca^2+^, 3 in the presence of Mg^2+^ ions, and 3 in the presence of Na^+^ ions. The highest increase in affinity due to the presence of divalent cations was found for complex number 5, and the only complex where the presence of divalent cations significantly reduced the HAS-HA affinity was complex number 6. These two complexes will be discussed again after considering Figure 5 and Figure 6.

In Figure 5a, the number of direct hydrogen bonds between HAS and hyaluronan is presented. The number of water bridges, where one water molecule forms a hydrogen bond to HAS and another one to hyaluronan are reported in Figure 5b. Another important piece of structural information, namely the number of ionic contacts and cation bridges when divalent cations are present, is summarized in Figure 6.

The binding energy distribution, Figure 4b, is quite different from the direct and water-mediated hydrogen bond distributions in Figure 5 and the distributions of ionic interactions reported in Figure 6. This directly shows that the binding affinity cannot be related to only one type of interaction, but rather is a complex function of many different types of interactions. However, when we consider the situation in the presence of Na^+^ ions we see that the most energetically favorable complexes, particularly complexes 1 and 2 but also complexes 3, 4, 6, 8 (Figure 4) are also the ones that display most direct hydrogen bonds and water mediated hydrogen bonds (Figure 5). This suggests that the dominant interactions in the albumin–hyaluronan system are hydrogen bonds in sodium containing solutions.

We now consider the more complex situation where divalent cations also are present. It is worth noting that, in general, proteins form more stable complexes with different species in the presence of divalent cations, rather than monovalent ones, as evidenced by various examples available in the literature, such as transcription activator-like effector proteins-DNA [52], E2 human papillomavirus regulatory protein-DNA [53] and anti-terminator protein-RNA [54]. The formation of a cationic bridge contributes to the improvement of a complex stability. In case of the HSA–HA system, the highest binding affinity is observed for complex 1 in presence of Ca^2+^ ions. Interestingly, this complex is characterized by a similar number of direct H-bonds as complex 2. These interactions are most abundant in these two complexes. However, taking into account a significantly lower binding energy in case of complex 2 comparing to complex 1 (Figure 4b), it can be concluded that the number of hydrogen bonds is not sufficient to describe the stability of the complex. Furthermore, in the case of complex 1, a low number of ionic contacts and divalent cation bridges can be found (Figure 6). On the other hand, complex 2 is characterized by a high number of water mediated H-bonds, when compared with other complexes formed in presence of Ca^2+^ ions. Complex 3 is also characterized by a high binding affinity in presence of Ca^2+^ ions. However, for this complex direct and water mediated H-bonds are relatively few, but the number of cation bridges are the highest (Figure 6b). Thus, here clearly the presence of Ca^2+^ ion mediated bridges is of importance for the high binding affinity.

As exemplified above, the situation is rather complex in the presence of divalent ions where the number of hydrogen bonds are affected as well as direct electrostatic interactions, and now cation bridges mediated by the divalent ions appear. For instance, complex 5 where the binding energy is most markedly increased in presence of divalent ions, we find that the presence of divalent ions increases both direct and water mediated H-bonds as well as ionic contacts. Calcium ion mediated bridges are also present. In contrast, in complex 6 where the presence of divalent ions reduces the binding affinity, we find a lower number of direct and water mediated H-bonds when the divalent ions are present. We note that in most complexes more cation bridges are formed with the less hydrated Ca^2+^ ion compared to the more hydrated Mg^2+^ ions. Interestingly, according to Vorum et al. [55] domain III, which is involved in forming all complexes indicated by the docking procedure (Table 1) is probably the key albumin Ca^2+^ binding site.

Pathological changes during osteoarthritis (OA) lead to Na^+^ and Ca^2+^ concentration increase [56]. Simultaneously, the concentration of HSA remains relatively unchanged, whereas HA concentration can decrease significantly (relative to other macromolecular components) [57]. Additionally, molecular mass of HA decreases [58]. As a result, the OA synovial fluid reveals much worse tribological properties. It was shown that increasing the HA concentration results in the gel point shift towards lower temperatures [59] in HAS–HA solutions. Based on this, binding between HA and HSA in OA synovial fluid will decrease (regardless of the increase in calcium ion concentration), and as a result, the complex will not be as stable, which can lead to poor lubrication as it will be diluted with water and ratios between components will change. Therefore, the direct introduction of calcium and hyaluronic acid (of high molecular weight) in relatively high concentrations into the joints (by injection) seems to be the most beneficial for their regeneration. Indeed, the HA supplementation has been widely used in treatment of joint diseases [60]. According to García-Padilla et al. (2015) [61], the injections of sodium bicarbonate and calcium gluconate solutions can be effectively used in knee osteoarthritis treatment. Interestingly, this effect is not pronounced when the oral supplementation is applied [62].

**Figure 7:**
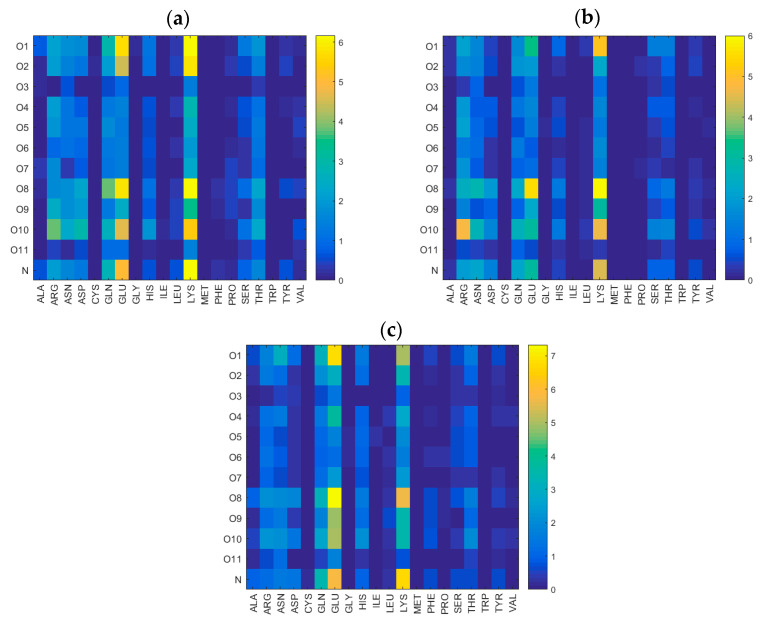
Hydrogen bond distribution between different oxygen classes in HA and different amino acids in HSA. Data were obtained in solutions containing (**a**) Na^+^, (**b**) Ca^2+^, (**c**) Mg^2+^. In all cases, Cl^−^ was the anion.

In Figure 7, the distribution of direct hydrogen bond sites between HSA and HA corresponding to different amino acids in HSA is presented, and the dominant sites are glutamine (GLU), and the cationic lysine (LYS). This is true in all three electrolyte solutions considered. However, hydrogen bonds between the cationic ARG and O10 atoms in hyaluronan were also found to be important in stabilizing the hyaluronan–albumin system in the presence of Ca^2+^ ions.

**Figure 8:**
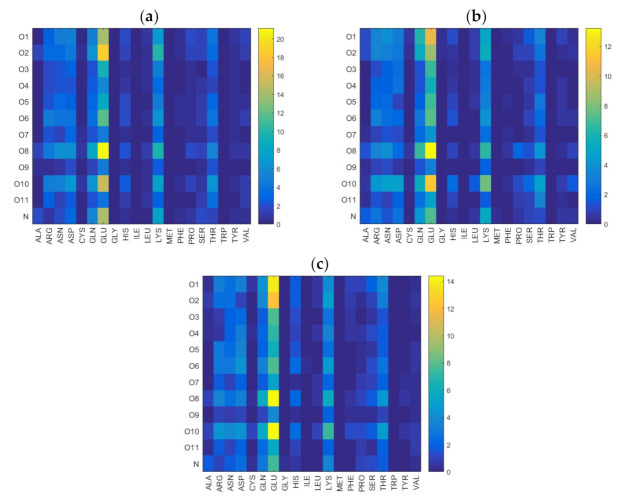
Water bridge distribution between different oxygen classes in HA and different amino acids in HSA. Data were obtained in solutions containing (**a**) Na^+^, (**b**) Ca^2+^, (**c**) Mg^2+^. In all cases, Cl^−^ was the anion.

**Figure 9:**
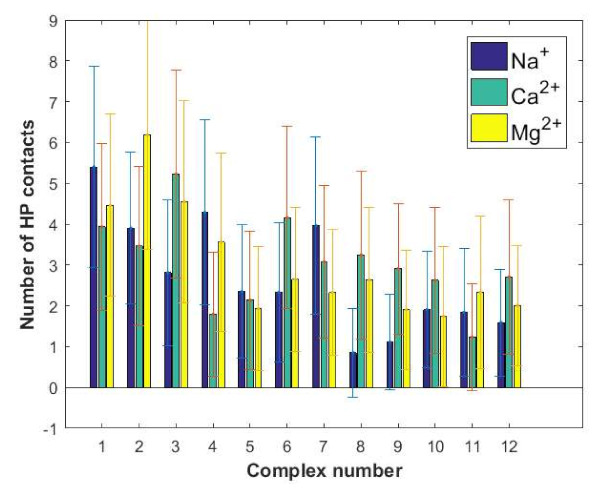
Number of hydrophobic interactions between HSA and HA.

Similar maps for water mediated hydrogen bonds are shown in Figure 8. Again, we find that most hydrogen bonds involve the GLU and LYS units in albumin, with a preference for GLU. In hyaluronan it is primarily the oxygen classes O1, O2, O8, and O10, containing OH-groups that can act as both H-bond donators and acceptors, as well as the amide nitrogen (also a H-bond donator and acceptor) that participate in hydrogen bonds, direct and water mediated, with albumin. Figure 9 shows the distribution of hydrophobic contacts between HSA and HA. The number of such contacts is relatively small, but we find a tendency of more hydrophobic contacts in the most energetically favored complexes. The amino acids that mainly contribute to this type of interactions are GLN, PRO, THR, HIS, and LYS.

## 3. Methods

To obtain the most stable complexes, we docked HSA–HA complexes using the VINA method [63] with default parameters and point charges initially assigned according to the AMBER14 force field [64] (the HA molecule was parametrized by applying the GLYCAM06 force field) and then damped to mimic the less polar Gasteiger charges used to optimize the AutoDock scoring function. The setup was done with the YASARA molecular modeling program [65,66]. The best hit of 50 runs with -10 kcal/mol free energy of binding was selected as distinctive complexes varying with the position of HA.

We obtained 12 complexes by varying the HA position as shown in Table S1 and in the Supplementary Materials. The albumin (PDB code:1e78) with hyaluronate simulation was run with YASARA. The setup included an optimization of the hydrogen bonding network [67] to increase the solute stability and a pKa prediction to fine-tune the protonation states of the protein residues at the chosen pH of 7.4 [68]. 70 Na^+^, Mg^2+^, Ca^2+^ ions, and sufficient Cl^−^ ions were added to achieve charge neutralization in the simulation cell. After steepest descent and simulated annealing minimizations to remove clashes, the simulation was run for 100 ns using the AMBER14 force field [69] for the HSA, GLYCAM06 [70] for HA, and TIP3P for water. The cut-off distance was set to 10 Å for van der Waals forces (the default used by AMBER [71]), no cut-off was applied to electrostatic forces (using the Particle Mesh Ewald algorithm, [72]). The equations of motions were integrated with multiple time steps of 1.25 fs for bonded interactions and 2.5 fs for non-bonded interactions at a temperature of 310 K and a pressure of 1 atm (NPT ensemble) using algorithms described in [68]. After inspection of the solute RMSD (root mean square deviation) as a function of simulation time, the first 40 ns were considered equilibration time and excluded from further analysis.

### 3.1. Binding Energy Calculation

The binding energy ($E_{bind}$) was calculated according to Equation (1). The high positive values of $E_{bind}$ denote high affinity of ligand to the protein [73]

(1) $$E_{bind}=E_{pot1}+E_{pot2}+E_{sol1}+E_{sol2}-(E_{pot-comp}+E_{sol-comp})$$

where *E*_pot1_ and *E*_pot2_ are potential energies of receptor and ligand, respectively, *E*_solv1_ and *E*_solv2_ are solvation energies of albumin and hyaluronan, respectively, *E*_pot-comp_ and *E*_sol-comp_ stand for the potential energy and solvation effects of the ligand–receptor system.

### 3.2. Hydrogen Bond Characteristics Determination

In this study, we followed the YASARA hydrogen bond (HB) geometrical and energetical features determination. According to this convention, the HB occurs when the hydrogen bond energy is higher than 6.25 kJ/moL. The distance between Hydrogen and Acceptor is correlated with the hydrogen bond energy (expressed in kJ/mol) according to eq. 2:

(2) $$E_{\mathrm{HB}}=25\cdot \frac{2.6-\max(Dis_{H-A},2.1)}{0.5}\cdot s_{D-A-H}\cdot s_{H-A-X}$$

$s_{D-A-H}$ stands for the first scalling factor and its value is related to the angle between donor, hydrogen, and acceptor, while the second scaling factor value ($s_{H-A-X}$) is dependent on the angle between hydrogen, acceptor, and the atom attached to the acceptor. These parameters can be calculated from Equation (3):

(3) $$S\left(\alpha \right)=\frac{\theta_{2}-\alpha }{\theta_{2}-\theta_{1}}$$

If the α parameter is lower than $\theta_{1}\;$or higher than $\theta_{2}$, scaling factors can be defined as follows:

(4) $$s_{H-A-X}=\left\{\begin{array}{c} 0,\\ S\left(\alpha \right),\\ 1, \end{array}\begin{array}{c} 0<x\leq \theta_{1}\\ \theta_{1}<x\leq \theta_{2}\\ \theta_{2}<x\leq 180{}^{\circ} \end{array}\right.$$

Depending on the atom type, $\theta_{1}$ and $\theta_{2}$ angles are different. For instance, in the case of heavy atoms$\;\theta_{1}$ and $\theta_{2}$ are 85° and 95°, respectively. On the other hand, in the case of hydrogens these angles are 75° and 85°.

### 3.3. Water Bridges

**Figure 10:**
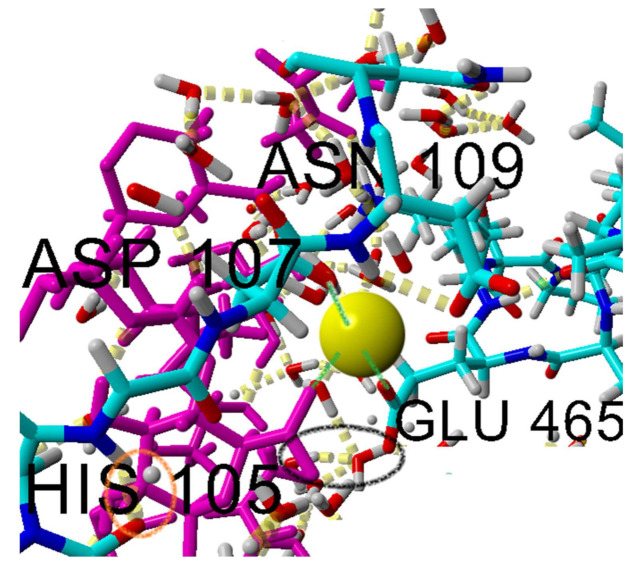
Water bridge visualization.

A water bridge (visualized in Figure 10) is formed is due to the interaction involving nitrogen or oxygen atoms, playing the accepting role and two hydrogen bonds donors in water molecule. According to the Yassara convention, the 3 Å distance was applied as a threshold in water bridge detecting.

### 3.4. Ionic Interactions

**Figure 11:**
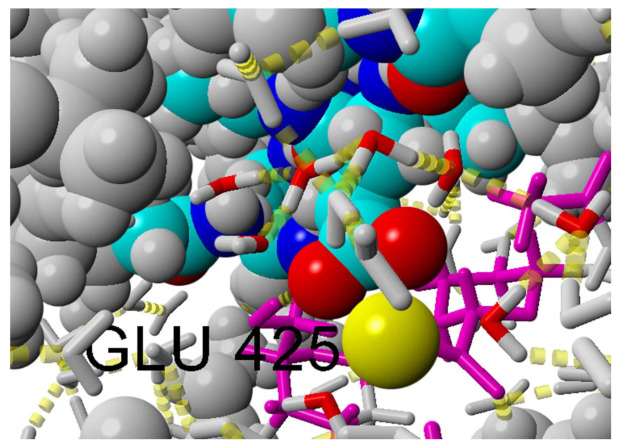
Cation bridge visualization.

Ionic interaction (visualized in Figure 11) is identified by calculating the distance between two atoms at the center of a formal integer charge of opposite signs (for LYS, this is simply the NZ atom with a formal charge of +1). Distance between two atoms is then subtracted with hydrogen bond radii, and in the range 0–1.5 A. Direct ionic contacts between HA and HSA occur between lysine and carboxyl groups in HA. Interestingly, we find no direct ionic contacts between carboxylate in HA and the positively charged HIS and ARG amino acids. This may suggest that the aromatic ring sterically hinders the cationic charge of HIS and that the diffuse charge of ARG, distributed between two amino groups, counteracts direct ionic contacts.

Cation bridges are defined as places at which a cation creates ionic interaction with both HSA and HA. These interactions occur between the carboxyl group of HA–ion (Ca, Mg) and negatively charged amino acids—glutamic acid (majority) and aspartic acid (rarely). This is due to the fact that the carboxylate group of glutamic acid resides further away from the polypeptide backbone.

### 3.5. Hydrophobic Interactions

The hydrophobic interactions are divided into three subcategories [56]:

− carbons carrying three hydrogen atoms,
− carbon carrying two hydrogen atoms or carbon with one hydrogen and three carbon atoms attached,
− carbons forming an aromatic ring carrying hydrogen atoms.

This definition excludes the Calpha atoms of amino acids (except Gly) since these are polar and can form weak hydrogen bonds.

## 4. Conclusions

In this study albumin–hyaluronan complexes, and in particular the effect of Na^+^, Ca^2+^, and Mg^2+^ ions, was investigated using molecular dynamics tools. In general, the research on the influence of ions on albumin binding capacity is interesting from a practical point of view. The appropriate proportion of ions provided by the proper supplementation of micronutrients can lead to stronger interactions between albumin and hyaluronate, which are two key components of synovial fluid. It was established that the divalent Ca^2+^ ions contribute mostly to the increase of the HSA affinity to HA. Moreover, most cation bridges are formed with Ca^2+^, whereas the effect of Mg^2+^ is less clear.

The bonding mechanism in the case of HSA–HA is associated with the presence of locally positively charged sites, amplified by the presence of divalent cations. These positive patches allow the hyaluronan to locally approach closely to the protein, which facilitates formation of HSA–HA hydrogen bonds. All probable binding sites were structurally and energetically characterized including solvation effects. Hydrogen bonds (direct and water mediated) are important for the complex formation. Hyaluronan mainly binds to the IIIA and IIIB domains of albumin. A more detailed analysis of the structure of the most preferred complex stabilized with calcium ions allowed us to observe a significant contribution of the amino acids GLU and LYS in the formation of intermolecular interactions.

## Figures and Tables

**Table 1:** The MD and docking ranks of potential HSA–hyaluronan complexes.

HSA–Hyaluronan Complex Number ^1^	HSA Binding Domains
1(2)	IA-IB-IIIA-IIIB
2(7)	IA-IIIA-IIIB
3(10)	IA-IIIA-IIIB
4(1)	IA-IB-IIIA-IIIB
5(5)	IIIA-IIIB
6(3)	IA-IIIA-IIIB
7(9)	IA-IIIA-IIIB
8(11)	IIIA-IIIB
9(12)	IIB-IIIA-IIIB
10(6)	IA-IIIA-IIIB
11(8)	IIIA-IIIB
12(4)	IIIA-IIIB

^1^ Ranking of obtained complexes. First number shows the rank after MD simulations, in the parentheses the rank of the structure according to docking procedure is presented.

## Data Availability

All data is available in the manuscript or upon request to the corresponding author.

## Supplementary Materials

The following are available online at https://www.mdpi.com/article/10.3390/ijms222212360/s1, Table S1. Distribution of amino acid contacts with HA. Ranking, Table S2. Distribution of amino acid (AA) in HSA and domain III.
