# Supplementary material for: Albumin–Hyaluronan Interactions: Influence of Ionic Composition Probed by Molecular Dynamics
Source: Int J Mol Sci. 2021 Nov 16;22(22):12360. doi: 10.3390/ijms222212360 (PMC8625520; doi:10.3390/ijms222212360)
Supplement: Supplementary file 1 [file ijms-22-12360-s001.zip › ijms-1437059-supplementary.pdf]

Supplementary Materials

Albumin – Hyaluronan interactions: influence of ionic composition probed by molecular dynamics

Piotr Bełdowski [1,2], Maciej Przybyłek [3], Przemysław Raczyński [4,] Andra Dedinaite[2,5], Krzysztof Górny [4,] D.C. Florian Wieland[6], Zbigniew Dendzik[4], Alina Sionkowska [7] and Per M. Claesson [8]

[1] Institute of Mathematics & Physics, UTP University of Science & Technology, 85-796 Bydgoszcz, Poland; piotr.beldowski@utp.edu.pl
[2] KTH Royal Institute of Technology, School of Engineering Sciences in Chemistry, Biotechnology and Health, Engineering Pedagogics, SE-100 44 Stockholm, Sweden.; andra@kth.se
[3] Department of Physical Chemistry, Pharmacy Faculty, Collegium Medicum of Bydgoszcz, Nicolaus Copernicus University in Toruń, Kurpińskiego 5, 85-950 Bydgoszcz, Poland; m.przybylek@cm.umk.pl
[4] University of Silesia in Katowice, Faculty of Science and Technology, 75 Pułku Piechoty 1A, 41-500 Chorzow, Poland; krzysztof.gorny@us.edu.pl (K.G); zbigniew.dendzik@us.edu.pl (Z.D.)
[5] RISE Research Institutes of Sweden, Division of Bioscience and Materials, SE-114 86 Stockholm, Sweden
[6] Helmholtz-Zentrum hereon: Institute for metallic Biomaterials, Max-Planck-Straße 1, 21502 Geesthacht, Ger-many
[7] Department of Biomaterials and Cosmetics Chemistry, Faculty of Chemistry, Nicolaus Copernicus University in Toruń, Gagarin 7, 87-100 Toruń, Poland; }; alinas@umk.pl
[8] KTH Royal Institute of Technology, School of Engineering Sciences in Chemistry, Biotechnology and Health, Deptment of Chemistry, Surface and Corrosion Science, SE-100 44 Stockholm, Sweden.; percl@kth.se

Table S1. Distribution of amino acid contacts with HA. Ranking

**Rank**	**1**		**2**		**3**		**4**		**5**		**6**		**7**		**8**		**9**		**10**		**11**		**12**	
	#	%	#	%	#	%	#	%	#	%	#	%	#	%	#	%	#	%	#	%	#	%	#	%
ALA	3	3.90	0	0.00	0	0.00	5	15.15	3	5.45	0	0.00	0	0.00	4	13.79	0	0.00	3	4.92	1	2.17	3	7.89
ARG	6	7.79	5	7.04	4	6.78	0	0.00	4	7.27	0	0.00	4	6.78	1	3.45	2	3.57	3	4.92	1	2.17	2	5.26
ASN	2	2.60	3	4.23	3	5.08	0	0.00	3	5.45	2	5.56	2	3.39	0	0.00	2	3.57	1	1.64	3	6.52	2	5.26
ASP	4	5.19	5	7.04	5	8.47	2	6.06	3	5.45	2	5.56	4	6.78	1	3.45	4	7.14	3	4.92	3	6.52	0	0.00
CYS	2	2.60	2	2.82	2	3.39	0	0.00	2	3.64	0	0.00	2	3.39	0	0.00	1	1.79	4	6.56	0	0.00	1	2.63
GLN	4	5.19	4	5.63	5	8.47	3	9.09	4	7.27	2	5.56	5	8.47	2	6.90	4	7.14	5	8.20	4	8.70	4	10.53
GLU	10	12.99	10	14.08	7	11.86	4	12.12	9	16.36	4	11.11	8	13.56	4	13.79	7	12.50	14	22.95	5	10.87	8	21.05
GLY	2	2.60	1	1.41	1	1.69	0	0.00	0	0.00	1	2.78	1	1.69	0	0.00	1	1.79	1	1.64	1	2.17	0	0.00

HIS	4	5.19	3	4.23	2	3.39	1	3.03	0	0.00	2	5.56	3	5.08	1	3.45	1	1.79	2	3.28	2	4.35	1	2.63
ILE	1	1.30	1	1.41	0	0.00	0	0.00	0	0.00	0	0.00	0	0.00	0	0.00	0	0.00	0	0.00	0	0.00	0	0.00
LEU	3	3.90	3	4.23	2	3.39	0	0.00	5	9.09	1	2.78	2	3.39	1	3.45	2	3.57	2	3.28	0	0.00	4	10.53
LYS	8	10.39	8	11.27	6	10.17	5	15.15	9	16.36	3	8.33	6	10.17	5	17.24	6	10.71	11	18.03	5	10.87	6	15.79
MET	0	0.00	0	0.00	1	1.69	0	0.00	0	0.00	1	2.78	0	0.00	0	0.00	1	1.79	0	0.00	1	2.17	0	0.00
PHE	4	5.19	3	4.23	2	3.39	1	3.03	0	0.00	1	2.78	3	5.08	1	3.45	3	5.36	2	3.28	1	2.17	0	0.00
PRO	5	6.49	6	8.45	5	8.47	3	9.09	3	5.45	4	11.11	5	8.47	3	10.34	6	10.71	3	4.92	5	10.87	3	7.89
SER	2	2.60	1	1.41	2	3.39	2	6.06	2	3.64	2	5.56	2	3.39	1	3.45	2	3.57	3	4.92	2	4.35	1	2.63
THR	10	12.99	10	14.08	9	15.25	2	6.06	6	10.91	6	16.67	9	15.25	2	6.90	8	14.29	0	0.00	7	15.22	1	2.63
TRP	0	0.00	0	0.00	0	0.00	0	0.00	0	0.00	0	0.00	0	0.00	0	0.00	0	0.00	0	0.00	0	0.00	0	0.00
TYR	2	2.60	3	4.23	2	3.39	1	3.03	0	0.00	2	5.56	2	3.39	1	3.45	2	3.57	3	4.92	2	4.35	0	0.00
VAL	5	6.49	3	4.23	1	1.69	4	12.12	2	3.64	3	8.33	1	1.69	2	6.90	4	7.14	1	1.64	3	6.52	2	5.26
Total	77		71		59		33		55		36		59		29		56		61		46		38	

Table S2. Distribution of amino acid (AA) in HSA and domain III.

AA	No in domain III	% of all AA in domain	No in HSA	% of all in HSA
ALA	17	9.34%	58	10.74%
ARG	7	3.85%	20	3.70%
ASN	5	2.75%	16	2.96%
ASP	8	4.40%	33	6.11%
CYS	11	6.04%	34	6.30%
GLN	7	3.85%	16	2.96%
GLU	17	9.34%	60	11.11%
GLY	4	2.20%	11	2.04%

HIS	4	2.20%	15	2.78%
ILE	2	1.10%	8	1.48%
LEU	17	9.34%	58	10.74%
LYS	22	12.09%	53	9.81%
MET	2	1.10%	6	1.11%
PHE	7	3.85%	28	5.19%
PRO	8	4.40%	22	4.07%
SER	9	4.95%	23	4.26%
THR	13	7.14%	25	4.63%
TRP	0	0.00%	1	0.19%
TYR	3	1.65%	18	3.33%
VAL	19	10.44%	35	6.48%
